# Association of Dopamine Transporter Gene (DAT1) 40 bp 3′ UTR VNTR Polymorphism (rs28363170) and Cannabis Use Disorder

**DOI:** 10.1177/11782218231163696

**Published:** 2023-03-31

**Authors:** Holiness SA Olasore, Akinniyi A Osuntoki, Olubunmi A Magbagbeola, Abdur-Rasheed B Awesu, Anthony A Olashore

**Affiliations:** 1 Department of Biochemistry, College of Medicine of the University of Lagos, Idi Araba, Lagos, Nigeria; 2 Federal Neuropsychiatric Hospital, Yaba, Lagos, Nigeria; 3 Department of Psychiatry, Nelson R Mandela School of Medicine, University of KwaZulu-Natal, Durban, South Africa; 4 Department of Psychiatry, University of Botswana, Gaborone, Botswana

**Keywords:** Dopamine transporter, gene polymorphism, VNTR, cannabis smoking

## Abstract

**Introduction:**

Cannabis remains the most widely used illicit drug among Nigerians, often associated with psychiatric disorders. Since genetic predisposition has been implicated in substance use disorders, we, therefore, aimed at finding out the relationship between dopamine transporter gene (DAT1) polymorphism and cannabis use disorder.

**Methods:**

We recruited 104 patients from a tertiary psychiatric facility in Lagos, Nigeria, who were diagnosed with cannabis use disorder according to ICD-10 and 96 non-smokers as a comparative group. The smokers were screened with Cannabis Use Disorder Identification Test (CUDIT), and cannabis dependence was assessed with the Severity of Dependence Scale (SDS). Genotyping was carried out for the 40 bp 3′ UTR VNTR of the DAT1 (rs28363170).

**Results:**

The frequencies of 9R/9R, 9R/10R, 10R/10R among non-smokers and smokers were 14 (14.3%), 25 (26.2%), 57 (59.5%) and 17 (16.3%), 54 (51.9%), 33 (31.7%) respectively. The genotype distribution was in Hardy Weinberg equilibrium (HWE) only in the smokers’ population (χ² = 1.896, *P* = .166). Individuals with the 10R allele were almost twice as likely as the 9R carriers to smoke cannabis (OR = 1.915, 95% CI: 1.225-2.995). However, this polymorphism was not associated with the quantity of cannabis smoked, age at onset of smoking, CUDIT, and SDS scores.

**Conclusion:**

The DAT VNTR polymorphism was associated with cannabis smoking but not cannabis use disorder.

## Introduction

Despite efforts globally to eradicate substance abuse, it remains a major health and social problem.^1^ Although cannabis has been removed from the list of illegal drugs in many jurisdictions, it is still the most prevalent illicit drug in use worldwide.^2^ Furthermore, Nigeria remains the country with the highest recorded use of cannabis.^3^ Cannabis dependence is the state of compulsive need for cannabis in which any form of harm (physical, mental, and social), as well as all other everyday interests, are ignored.^4^

The most important psychoactive substance in cannabis is the cannabinoid known as Δ^9^ tetrahydrocannabinol (THC), which produces the heightened feelings associated with the drug. This compound interacts with the cannabinoid receptor 1 (CB1) in the brain, whose natural ligand is the endocannabinoid neurotransmitter—anandamide.^5^ The brain’s mesolimbic pathway is believed to be chiefly involved in the brain reward pathway.^6^ The mesolimbic pathway connects the major dopamine-producing area in the brain, the ventral tegmental area (VTA), to the nucleus accumbens. This nucleus accumbens, an area in the ventral striatum, is linked with motivation and reward.^7^ Substances and habits, such as pathological gambling, associated with dependence are often found to increase the dopaminergic activities in the brain reward pathway.^8^

Apart from its effect on the cannabinoid receptor, THC has also been reported to have a great influence on the dopaminergic system, which may explain, at least in part, its addictive properties.^9^ The CB1 receptors are highly concentrated in the brain reward circuitry, where they may mediate in addiction. Dopaminergic neurons in this circuitry are regulated by excitatory and inhibitory inputs, which are controlled by the CB1 receptors and can be activated by THC.^10^ Once dopamine is released, it binds to the dopamine receptor subtypes (DRD1-DRD5). The action of dopamine released is terminated majorly by its metabolism and the dopamine transporter (DAT1), which is a key player in dopamine homeostasis, by mopping up dopamine after release. This transporter is a target for various drugs of addiction and dopamine reuptake inhibitors of therapeutic importance. Therefore, the dysfunction of this transporter is associated with neurotransmitter imbalances found in disorders that include drug addiction.^11^ The feeling of elation and the addiction caused by cocaine are believed to be majorly through the binding and blockade of the transporter.^12^

Genetic epidemiology studies especially using twin and adoption studies, have established a strong link between genetic risk factors and substance dependence.^13^ Genetic factors have been reported to account for 40% and 48% of the total variance in the initiation of cannabis use among women and men, respectively.^14^ In the cannabinoid receptor gene, some single nucleotide polymorphisms—the rs806368, rs806380, and rs1049353 have been found to be associated with cannabis dependence.^15-17^ The ABCB1 gene encoding P-glycoprotein is known to modify cannabinoid pharmacokinetics. The CC genotype of the rs1045642 polymorphism in this gene was independently associated with cannabis dependence.^18^

Since alterations in dopamine signaling have been implicated in drug addiction, including cannabis,^8,10^ polymorphisms of the genes in the dopamine signaling pathways are also expected to influence cannabis dependence. In one of our previous studies, we found that the A1 allele of the *Taq*IA polymorphism of the DRD2 was significantly associated with cannabis use disorder and dependence.^19^ Similarly, a significant association between the catechol-o-methyl transferase gene rs4680 polymorphism and cannabis use has also been reported.^20^

The dopamine transporter gene (DAT1) is a 68 kb gene that consists of 8 exons located on chromosome 5p15.32. Previous works have shown the relationship between the 40 bp 3′ untranslated region variable number of tandem repeats (3′ UTR VNTR) (rs28363170) of DAT1 gene and clinical phenotypes which are believed to be related to substance abuse such as cocaine-induced paranoia and methamphetamine-induced psychosis.^21,22^ Although Batalla et al^23^ have demonstrated that cannabis exposure alters the normal relationship between DAT1 polymorphism and the anatomy of total and subregional hippocampal volumes, we did not find any study relating this gene polymorphism to a tendency to abuse cannabis to the best of our knowledge. The present study, therefore, was focused on finding the relationship between the 3′ UTR 40 bp VNTR of the DAT1 gene and cannabis dependence.

## Participants and Methods

### Ethical approval and study participants

Before the study took off, we obtained the approval of the ethics committee of The Federal Neuropsychiatric Hospital, Yaba, Lagos, Nigeria. One hundred and four cannabis smokers attending the Drug Unit of the hospital who tested positive for urine toxicology screening were recruited. Questionnaires were given to the participants to obtain their biodata and data on cannabis use. The non-smokers were 96 healthy volunteers who were cannabis naïve and were negative for urine toxicology tests. All the participants agreed to participate in the study by signing an informed consent form.

### Cannabis use disorders screening

Screening for current use of cannabis was done using Cannabis Use Disorders Identification Test (CUDIT), which is a 10-item questionnaire according to DSM-IV.^24^

### Cannabis dependence assessment

Cannabis dependence was assessed using the Severity of Dependence Scale (SDS) by Adamson and Sellman.^25^

### Blood sample collection

About 1 ml of venous blood samples was taken from each of the participants in the smokers’ group in the morning following the day of admission and before breakfast. The same amount of blood samples was also taken from the volunteer non-smokers before breakfast. All blood samples were taken into EDTA sample bottles for the extraction of DNA.

### Extraction of DNA and genotyping

Genomic DNA was extracted from blood samples using a Blood DNA Preparation Kit (Jena Bioscience, Germany). Genotyping was carried out as previously described by Bhaskar et al^12^ with some modifications, using the following pair of primers: forward—5′-TGTGGTGTAGGGAACGGCCTGAG-3ʹ and reverse—5ʹ-CTTCCTGGAGGTCACGGCTCAAGG-3ʹ. The PCR reaction was performed with 5 μl of 5× Red Load Taq Master (Jena Bioscience, Germany) and 50 ng of template DNA, 2 μl each of reverse and forward primers. The reaction mixture volume was made up to 25 μl with PCR-grade water (Jena Bioscience, Germany). The PCR consisted of an initial 2 cycles of touchdown PCR cycles with a denaturation temperature of 94°C and 2 annealing temperatures of 66°C and 64°C each. These were then followed by 30 cycles of denaturation at 94°C for 30 seconds, annealing at 62°C for 1 minute, extension at 72°C for 1 minute, and final extension at 72°C for 2 minutes. The PCR product was afterward resolved on 2.5% agarose gel stained with GelGreen^®^ DNA stain (Biotium, USA). The expected fragments were 480 and 440 bp for 10 and 9 repeats, respectively.

### Statistical analyses

Statistical analyses were run on SPSS version 25. The distribution of the alleles between the smokers’ and non-smokers’ populations was compared using the Chi-square tests. This test was also used to estimate the relationship between the alleles and the cannabis use characteristics among the smokers and to estimate the Hardy-Weinberg equilibrium (HWE). Odds ratios were calculated by Woolf’s formula, while Fisher’s Exact Test was used to estimate the statistical significance, with values of *P* < .05 being considered significant. The descriptive statistics and the allele and genotype distributions concerning cannabis use parameters were carried out using frequency estimation.

## Results

The description of the participants in the smokers’ group is as presented (Table 1). The population genetics shows that the frequencies of the 9R and the 10R allele among the non-smokers were 53 (27.6%) and 139 (72.4%), while they were 88 (42.3%) and 120 (57.7%) among the smokers, respectively (Table 2). The genotype frequencies of the 9R/9R, 9R/10R, and 10R/10R among the non-smokers were 14 (14.3%), 25 (26.2%), 57 (59.5%), while for smokers, the frequencies were 17 (16.3%), 54 (51.9%), and 33 (31.7%) respectively. The genotype frequencies were not at HWE among the non-smokers (χ² = 9.554, df = 1, *P* = .002) but were in conformity with HWE among the smokers (χ² = 1.896, df = 1, *P* = .166). The 9R and 10R were the most common repeats. For the DAT VNTR polymorphisms, individuals with at least one 10R were almost twice as likely to use cannabis as those with the 9 repeats (*P* = .004, OR = 1.915, 95% CI = 1.225-2.995) (Table 3).

**Table 1.** Description of the smokers’ population.

	Frequency (n = 104)	Percent (%)
Age		
21-30	42	40.4
31-40	48	46.2
41-50	12	11.5
51-60	2	1.9
Marital status		
Single	57	54.8
Married	32	30.8
Separated/Divorced	15	14.4
Occupation		
Artisan	10	9.6
Road transport worker	3	2.9
Private employee/Business owner	32	30.8
Public servant	25	24.0
Student	22	21.2
Others	4	3.9
Unemployed	8	7.7
Age at first cannabis use		
10-14	16	15.4
15-19	49	47.1
20-24	24	23.1
25-29	6	5.8
30 and above	9	8.7
Quantity of cannabis used		
1 wrap	17	16.4
2-4 wraps	58	55.8
5-10 wraps	26	25.0
>10 wraps	3	2.9
Reason for cannabis use*		
Peers	87	83.7
Curiosity	6	5.8
Boredom	2	1.9
Advert	4	3.7
Other	5	4.9

* 1 wrap is equivalent to 5 g of dried cannabis leaves.

**Table 2.** Allele and genotype distributions among cannabis smokers and non-smokers.

	Non-smokers, n (%)			Smokers, n (%)		
Alleles	9R	10R		9R	10R	
	53 (27.6)	139 (72.4)		88 (42.3)	120 (57.7)	
Genotypes	9R/9R	9R/10R	10R/10R	9R/9R	9R/10R	10R/10R
	14 (14.3)	25 (26.2)	57 (59.5)	17 (16.3)	54 (51.9)	33 (31.7)
Hardy-Weinberg	χ^2^	df	*P*	χ^2^	df	*P*
	11.656	1.000	<.001	0.421	1.000	<.001

**Table 3.** Relationship between DAT 1 VNTR polymorphism and cannabis smoking.

Allele	Non-smokers	Smokers	χ^2^	*P*	OR	CI (95%)
DAT VNTR						
9R	53	88	9.457	.002	1.923	1.265-2.925
10R	139	120				

The results of the relationships between some cannabis smoking characteristics and the alleles as well as genotypes of the smokers are as presented (Table 4). The 9R allele was slightly more associated with cannabis dependence, although this was not significant (χ^2^ = 0.143, *P* = .413, OR = 1.123, CI (95%) = 0.614-2.055). The level of dependence was measured using the Severity of Dependence Scale (SDS). Most of the participants fell into the low/moderate dependence category. The 9R was more associated with low/moderate severity of dependence but also not significantly (χ^2^ = 0.527, *P* = .280, OR = 1.232, CI (95%) = 0.701-0.216). Cannabis use disorder was also assessed using the CUDIT, and it was found that most participants were in the low/moderate category. There was no significant association between the CUDIT scores and the various alleles (χ^2^ = 0.023, *P* = .496, OR = 0.958, CI (95%) = 0.552-1.663).

**Table 4.** Relationship between allele and genotype frequencies and some cannabis use characteristics of the smokers.

	Genotype, n (%)			Allele, n (%)		χ^2^	*P*	OR	CI (95%)
	9R/9R	9R/10R	10R/10R	9R	10R				
Dependence									
Yes	12 (70.6)	39 (72.2)	22 (66.7)	63 (71.6)	83 (69.2)	0.143	.413	1.123	0.614-2.055
No	5 (29.4)	15 (27.8)	11 (33.3)	25 (28.4)	37 (30.8)				
SDS									
Low/moderate	8 (47.1)	39 (72.2)	15 (45.5)	55 (62.5)	69 (57.5)	0.527	.280	1.232	0.701-0.216
High	9 (52.9)	15 (27.8)	18 (54.5)	33 (37.5)	51 (42.5)				
CUDIT									
Low/moderate	6 (35.3)	34 (63.0)	15 (45.5)	46 (52.3)	64 (53.3)	0.023	.496	0.958	0.552-1.663
High	11 (64.7)	20 (37.0)	18 (54.5)	42 (47.7)	56 (46.7)				
Family using cannabis									
Yes	10 (58.8)	36 (66.7)	23 (69.7)	56 (63.6)	82 (68.3)	0.502	.287	0.811	0.454-1.449
No	7 (41.2)	18 (33.3)	10 (30.3)	32 (36.4)	38 (31.7)				
Dose									
1-5 wraps	14 (82.4)	44 (81.5)	27 (81.8)	72 (81.8)	98 (81.7)	0.001	.563	1.010	0.496-2.059
>5 wraps	3 (17.7)	10 (18.5)	6 (18.2)	16 (18.2)	22 (8.4)				

Table 4 also shows that most participants had at least 1 family member who smoked cannabis. However, there was still no statistically significant relationship between the 2 alleles and having at least 1 family member smoking cannabis (χ^2^ = 0.502, *P* = .287, OR = 0.811, CI (95%) = 0.454-1.449). We found that most participants were smoking from 1 to 5 wraps (equivalent to 5 g of dried cannabis leaves) per day, but the analysis did not show any significant relationship between the quantity smoked and the alleles (χ^2^ = 0.001, *P* = .563, OR = 1.010, CI (95%) =  0.496-2.059).

## Discussion

Genetic factors are known major determinants of addiction, while dopamine is known to be involved in reward and motivation.^13,26^ Changes in dopaminergic signaling may therefore be expected to influence individual tendencies to addiction. We have, in this study, examined the relationships between a known polymorphism of the DAT1 gene coding for a key protein in the dopaminergic signaling pathway, cannabis use, and cannabis dependence among patients receiving treatment for cannabis use disorders at a tertiary treatment facility within Lagos in Nigeria.

The modal age of the smokers in this study was relatively higher than what was reported by most authors. This age difference was probably more due to the population sampled. It would be expected that where youth populations were targeted, such as secondary or tertiary institutions, the modal age could be lower, as expected.^27^ However, in a study conducted at a tertiary treatment facility for drug abusers, the mean age reported was closer to our finding.^28^ The same study by Effiong et al. also reported the mean age at the onset of smoking, which fell within the modal class for the age at smoking onset found in our study.

Most of the smokers in this study were single. Marital status has been associated with cannabis smoking, and having a marriage partner could influence smoking status as this could reduce the effect of boredom, peer pressure, and exposure to the lifestyle that could predispose to smoking.^29^ We found that most of the participants were either private employees or private business owners; this was contrary to what was expected in our environment, where cannabis smoking and other drug use are often most prevalent among road transport workers.^30^ Nevertheless, this could also be because people in this occupation, due to their lifestyle, are less likely to be brought to a drug treatment facility where we recruited our participants. Furthermore, in agreement with the findings of Adejoh et al.,^31^ peer pressure was the commonest reason for the initiation of cannabis smoking. The quantities of cannabis smoked by the participants we sampled were higher than those reported previously in a study conducted among male university students in Nigeria.^32^ These differences could be why our study participants were at the treatment facility.

We first estimated the allele and genotype frequencies of the DAT1 3′ UTR VNTR polymorphisms across the populations of non-smokers and smokers. There were very few other alleles like the 3R, 7R, and 8R found among the participants. However, because all of them existed in heterozygous form with the 10R, they were all classified with 10R. The allele and genotype frequencies we found in this study are comparable to those found in some African, European, and other populations, with the 10R being the major allele.^33-35^

In addition, after testing the genotype frequencies for conformation to the Hardy-Weinberg equilibrium (HWE) test, the genotype frequencies in the smokers’ population conformed to HWE, showing that the genotype frequencies have remained constant over time. This is similar to the finding by Bhaskar et al^36^ in a population with alcohol dependence. Contrary to the study by Bhaskar et al., who also reported that the genotype distribution within the control population conformed to the HWE, we found that the genotype frequencies within the non-smokers’ population deviated from HWE. Given the ethnic diversity of the Nigerian population, this deviation from the HWE might be due to interbreeding, while forces like population stratification and natural selection could also be responsible.^37^ We noted that the 10R allele was more associated with cannabis smoking. The odds ratio shows that the likelihood of smoking was almost twice with the 10R compared to the 9R. A study by Guo et al^38^ reported that the 10R allele was more associated with risky behavior, including problematic use of cannabis, alcohol, tobacco, cocaine, heroin, and other illegal drugs. Another study by Stolf et al^39^ also found that the 10R/10R genotype was significantly associated with crack cocaine use.

Drug addiction or dependence can be described as nonmedical self-administration of drugs with characteristics of intoxication or withdrawal symptoms. Addiction usually manifests with 3 main symptom categories: exaggerated substance use, social or lifestyle impairment, and risky substance use.^26^ Although the 10R was associated with cannabis smoking in this study, we found that among the smokers, there was almost no relationship between the allele distributions and cannabis dependence. Both 9R and 10R were distributed almost equally among those that were cannabis dependent and those that were not. To the best of our knowledge, no study has linked this DAT1 polymorphism to cannabis dependence. Nevertheless, some authors have reported that the 9R allele was associated with alcohol dependence,^36^ and alcohol-withdrawal symptoms, such as seizures and delirium tremens.^40^ Other reports have shown the contrary among tobacco smokers.^41,42^ While the mechanism of action of alcohol is mainly through GABA signaling,^43^ nicotine acts mainly by binding specific nicotinic acetylcholine receptors.^44^ Addiction to substances often involves dopamine signaling directly or indirectly. Although the THC in the cannabis through the cannabinoid receptor indirectly activates the dopaminergic system,^45^ the effect of the 9R allele might also be modulated by other genetic factors.

The DAT1 VNTR is located in the 3′ untranslated region of the transporter protein, and it does not affect the amino acid sequence. However, it is a functional polymorphism that affects the transporter protein density.^46^ Striatal dopamine is important for positive symptoms of substance use disorders like increased drug intake and craving; it also leads to impaired decision-making that underlies compulsive behavior, reduced sociality, and risk-taking.^26^ The 9R allele was associated with increased DAT activity in the studies on human adults using positron emission tomography (PET) or single-photon emission computed tomography (SPECT).^46^ The increase in the density of the DAT will expectedly lead to increased mopping up of dopamine back into the presynaptic neuron and thus the termination of dopamine action.^47^

In our results, the 9R allele was slightly more associated with lower severity than the 10R allele, although this was not statistically significant. A study reported that the 9R/9R genotype was protective against a spectrum of risky behaviors, such as substance abuse, compared to the 9R/10R and 10R/10R genotypes in young adulthood.^38^ Similarly, as noted above,^41,42^ the 9R has been described as protective against a high level of dependence among alcoholics and cigarette smokers. Besides, we did not observe any difference between the carriers of the 2 alleles and CUDIT scores, showing that the DAT 3′ VNTR polymorphisms were not associated with cannabis use disorder among the participants. It must be noted that the smokers were recruited from the psychiatric facility where they were being treated for substance use disorder. It is, therefore, not surprising that most of them met the criteria for cannabis dependence and cannabis use disorder.

We found that most participants in this study had family members who used cannabis, and they also started using cannabis at an early age. Smokers with the 10R allele were more likely to have a family member using cannabis than the carriers of the 9R allele, although this difference was also insignificant. Likewise, no pattern in our results suggested any of the alleles could predict the age at the onset of cannabis use, although most participants with the 10R alleles tended to start smoking below the age of 20. Besides, the results did not also show that any of the 2 major alleles were predictive of the quantity of cannabis used by the participants. All these may be because drug use often has complex etiology involving many genes interacting with one another and social and environmental factors.

## Conclusion

In conclusion, we observed that the DAT 3′ UTR VNTR polymorphism influences cannabis use. While having a 10R could predict who would smoke cannabis, none of these alleles was found to be predictive of cannabis dependence despite most participants having some level of cannabis dependence. However, the 9R was found to be associated with lower severity of dependence. We also could not find an association between these alleles and cannabis use disorder, age at onset of cannabis use, and quantity of cannabis used. Future studies will benefit from a larger sample size to increase the study’s statistical power. It may also be beneficial to use a cross-section of smokers not within any rehabilitation facility for the study to be more inclusive. Doing this may include female smokers as well, as none of the participants from the psychiatry facility was female.
